# The IARC Perspective on the Effects of Alcohol Policies on Reducing Alcoholic Beverage Consumption

**DOI:** 10.1056/NEJMsr2413289

**Published:** 2025-05-01

**Authors:** Susan M. Gapstur, Daniela Mariosa, Luciana Neamtiu, Suzanne T. Nethan, Jürgen Rehm, Taisia Huckle, David H. Jernigan, Elizabeth A. O’Connor, Mindaugas Štelemėkas, Peter Allebeck, Sawitri Assanangkornchai, Nicholas Carah, Surasak Chaiyasong, Samantha Cukier, The Son Dao, Rijo M. John, Richard Matzopoulos, Petra S. Meier, Paula O’Brien, Guillermo Paraje, Robin Room, Claire Wilkinson, Norman D. Maldonado, Tim Stockwell, Maria Neufeld, Dag Rekve, Béatrice Lauby-Secretan

**Affiliations:** 1 International Agency for Research on Cancer, Lyon, France; 2 Centre for Addiction and Mental Health, Toronto, ON, Canada; 3 Centre for Social and Health Outcomes Research and Evaluation and Whariki Research Centre, Massey University, Auckland, New Zealand; 4 Boston University School of Public Health, Boston University, Boston, MA, USA; 5 Kaiser Permanente Center for Health Research, Portland, OR, USA; 6 Lithuanian University of Health Sciences, Kaunas, Lithuania; 7 Department of Global Public Health, Karolinska Institutet, Solna, Sweden; 8 Prince of Songkla University, Hat Yai, Songkhla, Thailand; 9 The University of Queensland, Brisbane, Queensland, Australia; 10 Mahasarakham University, Kantharawichai, Maha Sarakham, Thailand; 11 Public Health Agency of Canada, Ottawa, Ontario, Canada; 12 Center for Economics and Community Development, Hanoi, Vietnam; 13 Rajagiri College of Social Sciences, Kalamassery, Kerala, India; 14 Burden of Disease Research Unit, South African Medical Research Council, Tygerberg; 15 University of Cape Town, Division of Public Health Medicine Cape Town, South Africa; 16 University of Glasgow, Glasgow, Scotland, United Kingdom; 17 Melbourne Law School, The University of Melbourne, Melbourne, Victoria, Australia; 18 Business School, Universidad Adolfo Ibáñez, Peñalolén, Santiago de Chile, Chile; 19 Centre for Alcohol Policy Research, La Trobe University, Bundoora, Victoria, Australia; 20 University of New South Wales, Sydney, New South Wales, Australia; 21 PROESA – Research Center on Health Economics and Social Protection, Universidad Icesi, Cali, Colombia; 22 Canadian Institute for Substance Use Research, University of Victoria, Victoria, British Columbia, Canada; 23 World Health Organization Regional Office for Europe, Copenhagen, Denmark; 24 World Health Organization, Geneva, Switzerland

## INTRODUCTION

The International Agency for Research on Cancer (IARC) has classified alcoholic beverages as carcinogenic to humans (Group 1) based on *sufficient evidence* of causality for seven types of cancer (oral cavity, pharynx, larynx, esophagus, liver, colorectum, female breast).^1^ Globally, an estimated 741,300 (4.1%) new cancer cases in 2020 were attributable to alcohol consumption.^2^ In addition to cancer, alcohol consumption is a risk factor for numerous other health conditions.

**Figure 1.**
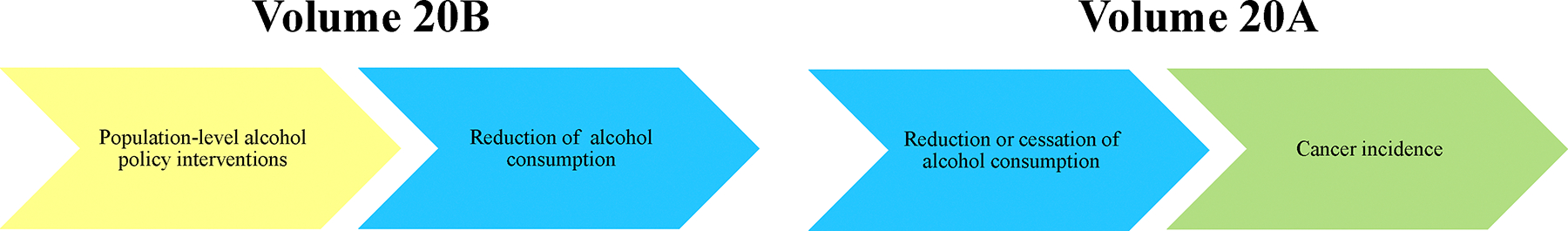
Schema for the development of *IARC Handbooks* Volumes 20A and 20B. This figure is updated from *IARC Handbooks* Volume 20A.^5^ Panel A shows the two-step evaluative framework from which, for scientific reasons, the level of evidence that an intervention prevents cancer is established by way of an intermediate outcome, as described in the *IARC Handbooks of Cancer Prevention* Preamble for Primary Prevention.^4^ In this framework, the intermediate outcome is defined as a change in exposure (decrease in exposure to an established cancer risk factor or increase in exposure to a cancer-preventive factor). In Step 1, the effect of a specified intervention on the intermediate outcome is evaluated. In Step 2, the effect of the intermediate outcome on cancer incidence in humans is evaluated. If it has not yet been established in authoritative sources that the intermediate outcome reduces cancer incidence in humans, Step 2 is conducted before Step 1. Panel B shows the development of *IARC Handbooks* Volumes 20A and 20B. Because the evidence that reduction or cessation of alcohol consumption reduces cancer incidence in humans had not been evaluated before, such evidence was reviewed and evaluated first.^5^ Then, the evidence that selected population-level alcohol policy interventions reduce alcohol consumption was reviewed and evaluated (*IARC Handbooks* Volume 20B, in preparation).

The *IARC Handbooks of Cancer Prevention* Program developed a two-part volume (20A and 20B) on the primary prevention of alcohol-related cancers (Figure 1).^5^

There is *sufficient evidence* that, compared with continuing consumption, reduction or cessation of alcoholic beverage consumption reduces risk of oral and esophageal cancers, and *sufficient evidence* from mechanistic studies that cessation of alcohol consumption reduces alcohol-related carcinogenesis (volume 20A).^5,6^

For Volume 20B, a Working Group reviewed and qualitatively evaluated the strength of the evidence on the potential for select alcohol policy interventions to reduce alcohol consumption (Figure). The interventions evaluated were selected because their implementation aims to reduce consumption at the national or subnational level, which has been shown to effectively reduce alcohol-attributable harms.^7^ In addition, the evidence on the effectiveness of healthcare-based interventions to reduce alcohol consumption is summarized but not evaluated, because they target individuals and their potential population-level effects on consumption are not usually measured. However, a robust body of evidence supports the use of screening, brief interventions,^8^ and longer-term psychosocial therapies,^9^ which may be combined with pharmacotherapy,^10^ to increase abstinence rates and improve other outcomes among individuals with alcohol use disorders. The key findings for Volume 20B are summarized herein.

## GENERAL METHODOLOGICAL CONSIDERATIONS

Only published studies with empirical data on selected alcohol policy interventions in relation to change in alcohol consumption – defined here as the sum of at least the three major alcoholic beverage types (i.e., beer, wine, and spirits) or each of those three types separately – were included (Table 1). The studies described below are those that provide the strongest evidence and/or geographical diversity. Unless otherwise specified, change in alcohol consumption refers to a change (or average difference) over time due to a restrictive or permissive alcohol policy intervention in (1) total or recorded average annual per capita consumption or sales in population-level studies, or (2) self-reported daily consumption in individual-level studies. In studies where the outcome in a regression model was expressed as the logarithm of alcohol consumption, the Working Group calculated the estimated percentage change due to the intervention.^11^

Exceptionally, for alcohol policy interventions with a paucity of studies with alcohol consumption outcomes, studies using proxy outcomes for alcohol consumption (e.g., liver cirrhosis mortality) were included if an association with alcohol consumption was previously established.

Several general methodological issues were considered when assessing study quality. These issues include control for important confounding factors (e.g., income), inclusion of appropriate control groups or jurisdictions, and in individual-level studies, evaluation of differential sampling, non-response, or reporting of alcohol consumption.

## TAX AND PRICE POLICIES

Taxes, minimum pricing, and bans or other restrictions on price promotions or quantity discounts are recommended interventions to reduce alcohol-related harm.^3^ Studies were included if the policy intervention was on the tax or price of at least one major alcoholic beverage type (i.e., beer, wine, or spirits). For tax specific policies, only studies that controlled for income or its proxies were included to ensure that the effect of the tax on consumption reflects the true net effect by accounting for affordability of alcohol.

Alcohol taxes in relation to alcohol consumption were assessed in seven studies. Increases in alcohol taxes were consistently associated with reductions in alcohol consumption or sales. The magnitude of the effect varied by context and by tax policy. In one of the most informative studies, a 2009 excise tax increase (21% for beer and 90% each for wine and spirits) in Illinois, USA, led to a greater decrease in monthly total ethanol purchases between the 24 months before and the 24 months after the tax increase compared with other USA states (−6.4%; *P*<0.05 among heavy-drinking households, and −5.0%; *P*<0.05 among higher-income households).^12^ Similarly, a 2011 alcoholic beverage sales 50% tax increase in Maryland, USA, led to a reduction in monthly per capita alcohol sales (−3.78%; 95% confidence interval [CI], −4.82% to −2.74%).^13^ The effect of the 2008–2009 Australian 70% excise tax increase on ready-to-drink beverages was assessed using annual individual-level data from 2002 through 2018;^14^ in a difference-in-difference model, by 2018, an 8.89% (*P*<0.001) reduction in average daily number of drinks consumed was observed. These results are supported by definitive evidence that an increase in taxation is passed through to prices and the ensuing increase in alcohol prices reduces alcohol consumption.^15^ The Working Group concluded that there is *sufficient evidence* that increases in alcohol taxes that increase prices lead to a reduction in alcoholic beverage consumption.

Minimum pricing policies — which include a minimum unit price (floor price for a fixed volume of alcohol) or a minimum price (floor price for a finished product) — in relation to alcohol consumption have been assessed in 10 studies conducted in Australia, Canada, and the United Kingdom (UK). Most studies assessed the May 2018 implementation of a minimum price of 50 pence per UK unit of alcohol in Scotland, UK; in the most recent, well-controlled population-level study, a net reduction (−3%; 95% CI, −4.2 to −1.8%) in total per adult alcohol sales was noted after 3 years of implementation.^16^ In an individual-level study of the same intervention, a 7.6% (*P*<0.05) decrease in grams of ethanol per adult per household purchased weekly was observed after 8 months of implementation.^17^ Overall, a reduction in alcohol consumption was consistently observed in studies that used different designs, analytical approaches, and sociodemographically defined groups. Therefore, the Working Group concluded that there is *sufficient evidence* that minimum pricing leads to a reduction in alcoholic beverage consumption.

Bans on alcohol discounts in relation to alcohol consumption were assessed in three studies, all of which assessed the same 2011 partial ban on multibuy discount of off-premises alcohol in Scotland, UK. In a study with population-level data, the ban led to a decrease (−1.7%; 95% CI, −3.1% to −0.3%) in off-premises per capita alcohol sales volume.^18^ In one study with individual-level data, an 8.6% (*P*<0.001) increase in weekly alcohol purchases was observed in the year after the ban,^19^ whereas in another study, which used overlapping data but different methods, no difference was observed between pre- and post-ban weekly alcohol purchases.^20^ Because of these inconsistencies, the Working Group concluded that there is *inadequate evidence* that bans on discounts of alcoholic beverages lead to a reduction in alcoholic beverage consumption.

## AVAILABILITY POLICIES

When alcoholic beverages are readily available, consumption increases.^7^ Studies that assessed availability policies that regulate alcohol outlet density, days or hours of sale, minimum purchase or drinking age, or prohibit alcohol sales in relation to alcohol consumption or proxy outcomes were included.

Policies regulating alcohol outlet density in relation to alcohol consumption or proxy outcomes were assessed in nine studies; restrictive policies were assessed in three of these studies. For example, restricting retail sales of alcohol in one community was associated with an approximately 2-fold greater reduction (*P* not reported) in violent crimes after (2003–2007) compared with before (1997–2002) the intervention relative to comparison communities.^21^ In a study with population-level data (2007–2015) in England, UK, the strictest local alcohol licensing policies were associated with 5% lower (*P*<0.05) annual alcohol-related hospital admissions compared with no licensing policy.^22^ In most studies of policy changes that increased outlet density, increases in alcohol consumption, assault rates, alcohol-related deaths, or alcohol-related hospital admission rates were observed. Because high-quality studies showed an inverse association between restrictions on density and alcohol consumption or proxy outcomes, and confounding could be ruled out with reasonable confidence, the Working Group concluded that there is *sufficient evidence* that restrictions on density of alcohol outlets lead to a reduction in alcoholic beverage consumption.

Six studies assessed alcohol policies regulating days of sale and five studies assessed policies regulating hours of sale in relation to alcohol consumption. All but one study were in high-income countries. In the only controlled trial, which was conducted from February 2000 to June 2001 in selected regions in Sweden, permitting alcohol sales on Saturdays in addition to weekdays increased average monthly per capita ethanol sales by 3.7% (*P*<0.001).^23^ In the largest individual-level study (using 2000–2019 annual surveys), state-level off-premises Sunday alcohol sales bans in the USA were associated with decreased alcohol consumption among women (−0.09 g ethanol per day; 95% CI, −0.10 to −0.08) and men (−0.04 g ethanol per day; 95% CI, −0.05 to −0.03).^24^ In a study with population-level data from Australia (1901–2006), earlier closing of on-premises outlets was associated with lower consumption (−2.9 liters of ethanol per capita per year, 95% CI, −3.4 to −2.4).^25^ Overall, in all studies, changes or average differences over time in hours or days of sale were associated with consumption in the expected direction; several high-quality studies were included, and all studies included a control group or controlled for confounding factors. Therefore, the Working Group concluded that there is *sufficient evidence* that restrictions on days or hours of sale lead to a reduction in alcoholic beverage consumption.

Minimum legal purchase or drinking age policies in relation to alcohol consumption were assessed in 10 studies in the USA and four studies in Europe or Canada. In most studies that assessed increases in minimum purchase or drinking age, reductions in alcohol consumption were observed. For example, in one study with population-level data (1961–2008) from five European countries, a 1-year increase in the minimum purchase age was associated with a decrease (−9.8%; 90% CI, −15.4% to −4.2%) in alcohol consumption.^26^ Similarly, the 1982 minimum purchase age increase from 18 to 19 years in New York, USA, was associated with a 21% (*P*<0.01) decrease in the prevalence of alcohol consumption among 18-year-old individuals.^27^ In most studies of decreases in minimum purchase or drinking age, observed associations with alcohol consumption were in the expected direction. Because the direction of the effects was consistent with the policy changes in most studies, the effect was observed on the specific age group targeted by the policy, and potential confounding by other alcohol availability policies was ruled out in the strongest studies, the Working Group concluded that there is *sufficient evidence* that increases in the minimum legal alcohol purchase or drinking age leads to a reduction in alcoholic beverage consumption.

Total bans on alcohol sales in relation to alcohol consumption or proxy outcomes were assessed in nine studies. In one study, the 1920–1935 USA National Prohibition of alcohol led to an initial 70% decrease in alcohol consumption, which eventually plateaued at a 30% to 40% decrease relative to pre-Prohibition levels.^28^ In a study in India, among men, the 2015 alcohol sales ban in one state resulted in a decrease in the weekly prevalence of alcohol consumption from 15.0% in 2015–2016 to 7.8% in 2020–2021, but not in neighboring states without a ban (adjusted difference in the change in prevalence, −7.1 percentage points; 95% CI, −9.6 to −4.6).^29^ In all other studies, a ban on sales also was associated with substantially lower alcohol consumption, prevalence of hazardous drinking, alcohol-related mortality, injury-induced mortality, and trauma admission rates. Because consistently large effects were observed in all studies across a range of settings, the Working Group concluded that there is *sufficient evidence* that bans on alcohol sales lead to a reduction in alcoholic beverage consumption.

## MARKETING BANS

Exposure to and/or engagement with alcohol marketing generally increases alcohol consumption, and among youth, a causal association has been established.^7^ Approaches for regulating alcohol marketing range from no restrictions to industry self- or co-regulation, to partial restrictions, to comprehensive bans on all forms of alcohol marketing in all media types and for all alcoholic beverage types.^30^

We did not identify any informative studies that assessed comprehensive alcohol marketing bans in relation to alcohol consumption. However, we found four informative population-level studies of strong bans – defined here as bans on alcohol marketing in at least one major media type (i.e., print, broadcast, or outdoor) for all alcoholic beverage types. In a study in Norway (1960–2006), the 1975 ban on marketing in all media types for all beverages with an alcohol content >2.5% was associated with a 7.1% (*P*=0.002) reduction in alcohol consumption.^31^ In two studies, based on data from 20 (1970–1995) and 17 (1975–2000) Organisation for Economic Co-operation and Development countries, average differences in alcohol consumption over time were compared between countries with and countries without strong bans.^32,33^ In one of these studies, a 1-unit increase in a “total alcohol advertising bans” score was associated with 8.6% (*P*<0.10) lower alcohol consumption.^32^ In the other study, bans on advertising in broadcast media for all alcoholic beverages except weak beer (~2.5% or less) were associated with 3.8% (*P*<0.05) lower alcohol consumption.^33^ The 14-month (September 1971–October 1972) strong alcohol marketing ban in one Canadian province resulted in no difference in alcohol consumption during the ban years (1971–1972) compared with the pre-ban years (1962–1970) (*z*=0.31, *P*>0.05), whereas consumption increased during the same time period in a comparable province without a ban (*z*=1.87, *P*<0.03).^34^ Because of the consistency of the evidence, the strength of the associations, and the fact that confounding could be ruled out with reasonable confidence, the Working Group concluded that there is *sufficient evidence* that strong bans on alcohol marketing lead to a reduction in alcoholic beverage consumption.

## COORDINATED MULTIPLE ALCOHOL POLICY INTERVENTIONS

Coordinated multiple alcohol policy interventions are defined here as government alcohol monopolies or a set of interventions enacted and implemented as part of a national action plan to reduce alcohol-related harm.

Government alcohol monopolies are integrated into national structures that can impose high alcohol taxes, restrict or ban promotional pricing strategies, limit availability, and restrict or ban marketing.^7^ In recent decades, few government alcohol monopolies have been established. The weakening or dissolution of a government alcohol monopoly in relation to alcohol consumption was assessed in 13 studies. In a study with population-level data from Poland (1961–2008), dissolution of the government alcohol monopoly in 1990 was associated with a 9% (*P*<0.10) increase in alcohol consumption.^35^ In a study with individual-level data, the 2012 Washington, USA privatization of liquor stores was associated with a 26% (*P*<0.05) increase in off-premises monthly alcohol purchases per household.^36^ In most other studies, de-monopolization of retail sales of one or all alcoholic beverage types was associated with higher alcohol consumption or higher prevalence of heavy drinking.

Studies of other coordinated multiple alcohol policy interventions that were implemented as part of national action plans in Thailand, Russia, Estonia, and Lithuania suggest a beneficial effect of multiple interventions on reducing alcohol consumption or the rate of increase in consumption.^7,37^

Because the evidence from population-level and individual-level studies was consistent and confounding by income and other factors could be ruled out with reasonable confidence, the Working Group concluded that there is *sufficient evidence* that government monopolies or other coordinated multiple alcohol policy interventions lead to a reduction in alcoholic beverage consumption.

## DISCUSSION AND CONCLUSION

Alcoholic beverages are established human carcinogens^1^ and, compared with continuing consumption, reduction or cessation of alcoholic beverage consumption reduces oral cancer and esophageal cancer risk.^5,6^ Based on the evidence reviewed, the Working Group concluded that the following alcohol policy interventions lead to a reduction in alcohol consumption: interventions that increase taxes, minimum prices, minimum alcohol purchase or drinking age; interventions that reduce alcohol outlet density and days or hours of sale; strong alcohol marketing bans; total bans on sales; and government monopolies or other coordinated multiple alcohol policy interventions.

Several issues relevant to the evaluations must be considered. First, the studies of alcohol availability interventions and marketing bans are almost entirely based on data collected before the expansion of digital marketing and online purchasing, and their relevance to related interventions in the current digital era may have limits. Second, most studies were conducted in high-income countries, and the effectiveness of some interventions may differ in other settings. Third, the evaluations focused on the effectiveness of alcohol policy interventions; examining their potential unintended consequences might inform feasibility in specific contexts but should not detract from the proven public health benefits. Finally, to be more effective, any policy must be enacted, implemented, and enforced, which requires the involvement of many different governmental and non-governmental sectors.

In conclusion, a substantial body of evidence exists on the effects of alcohol policies in reducing alcohol-related harm.^38^ Our findings add to the available evidence on the potential for alcohol policy interventions to reduce the alcohol-attributable cancer burden.^39^ Furthermore, they support the recommended actions stated in the most recent WHO Global Alcohol Action Plan 2022–2030,^40^ which includes the “SAFER” initiative*, and emphasize high impact strategies and interventions to reduce alcohol-related harm worldwide.

## Figures and Tables

**Table 1.** Assessment of the Strength of the Evidence of Selected Alcohol Policies in Relation to Alcoholic Beverage Consumption

Alcohol Policy	Number and Type of Studies	Strength of the Evidence*

***Tax and Price Policies***		
Excise and sales tax	4 Studies with population-level data 2 Studies with individual-level data 1 Study with both population-level and individual-level data	Sufficient
Minimum pricing	4 Studies with population-level data 6 Studies with individual-level data	Sufficient
Bans on discounts	1 Study with population-level data 2 Studies with individual-level data	Inadequate
***Availability Policies***		
Outlet density (n=6^†^)	8 Studies with population-level data (6 used proxy outcomes^‡^) 1 Study with individual-level data	Sufficient
Days or hours of sale (n=4^†^)	*Days of sale* 2 Studies with population-level data 4 Studies with individual-level data *Hours of sale* 4 Studies with population-level data 1 Study with individual-level data	Sufficient
Minimum purchase or drinking age (n=10^†^)	5 Studies with population-level data 9 Studies with individual-level data	Sufficient
Total bans on sales	7 Studies with population-level data^§^ (5 used proxy outcomes^‡^) 2 Studies with individual-level data^ǁ^	Sufficient
***Marketing Policies***		
Strong alcohol marketing bans	4 Studies with population-level data	Sufficient
***Coordinated Multiple Alcohol Policy Interventions***	*Government monopolies*^¶^ 10 Studies with population-level data 2 Studies with individual-level data 1 Study with both population-level and individual-level data *Other coordinated multiple alcohol policy interventions* 7 Studies with population-level data 3 Studies with individual-level data	Sufficient

* According to the criteria described in the *IARC Handbooks* Preamble for Primary Prevention,^4^ “sufficient evidence” indicates that a causal preventive association between the intervention and alcohol consumption (i.e., the intermediate outcome) in humans has been established; “limited evidence” indicates that a causal preventive association between the intervention and alcohol consumption in humans is plausible; and “inadequate evidence” indicates that the current body of evidence does not enable a conclusion to be drawn about the presence or absence of a preventive association between the intervention and alcohol consumption in humans.

† n indicates the number of studies that assessed a permissive policy change (i.e., increased outlet density, increased days or hours of sales, decreased minimum purchase and drinking age).

‡ Proxy outcomes for alcohol consumption included deaths (or mortality rates) from alcohol poisoning, liver cirrhosis, or injury; incidence rates of (or a proximity index to) violent crime or assault; rates of trauma hospital admission, and total alcohol-related hospital admission or mortality rates.

§ In one study, alcohol consumption was estimated using liver cirrhosis or alcoholism mortality rates, first hospital admission for alcoholic psychosis per capita, and drunk arrest rates.

ǁ In one study, alcohol consumption was reported at the time of the survey and recalled before the intervention.

¶ All 13 studies assessed the weakening or dissolution of a government alcohol monopoly.

## Footnotes

* Footnote SAFER is an acronym for methods to reduce alcohol-related harm: S-- Strengthen restrictions on alcohol availability A-- Advance and enforce drunk driving counter measures F-- Facilitate access to screening, brief interventions and treatment E-- Enforce bans or comprehensive restrictions on alcohol advertising, sponsorship, and promotion R-- Raise prices on alcohol through excise taxes and pricing policies
